# Effectiveness of the Attachment Design and Thickness of Clear Aligners during Orthodontic Anterior Retraction: Finite Element Analysis

**DOI:** 10.1055/s-0043-1761452

**Published:** 2023-03-24

**Authors:** Ananto Ali Alhasyimi, Aulia Ayub, Cendrawasih Andusyana Farmasyanti

**Affiliations:** 1 Department of Orthodontics, Faculty of Dentistry, Universitas Gadjah Mada, Yogyakarta, Indonesia

**Keywords:** attachment, clear aligner, force, orthodontic, retraction, thickness

## Abstract

**Objective** Clear aligner treatment (CAT) provides orthodontic patients with a comfortable treatment alternative; however, this device has limited capacity to facilitate tooth movements. Although composite attachment has been proposed to facilitate tooth displacement, some of its aspects, such as aligner thickness, can influence CAT's precision. This work aimed to compare the stress distribution patterns produced by clear aligners with different thicknesses and composite attachment shapes during anterior retraction.

**Materials and Methods** Maxillary models consisting of clear aligners, maxillary teeth, and various attachments to the upper central incisor's labial surface were generated. Three models were built to mimic the retraction of the upper central incisors. Each had a distinct attachment design (rectangular attachment, ellipsoid attachment, and pyramidal attachment) and various aligner thicknesses (0.75, 0.85, 0.95, 1.05, and 1.15 mm). Upper central incisor retraction was accomplished using clear aligners. Finite element analysis was used to examine the built models. Stress distribution pattern was examined.

**Results** The greater the thickness of the aligner, the higher the stress experienced by the teeth. The 0.75 mm-thick aligner induces the lightest stress with a minimum of 0.0037623 MPa and a maximum of 0.32859 MPa. Meanwhile, the 1.5 mm-thick aligner has the highest stress with a minimum of 0.004679 MPa and a maximum of 0.43858 MPa. The force distribution on rectangular attachments appears evenly distributed. The maximum pressure force on rectangular attachments has a minimum of 0.38828 MPa, which is smaller than the maximum on ellipsoid and pyramidal attachments at 0.40933 and 0.45099 MPa, respectively.

**Conclusion** The best aligner thickness is 0.75 to 0.85 mm for anterior retraction. An aligner with 0.95 mm thickness can still be used when a remarkable amount of tooth movement force is needed; however, this exception is only applicable to a limited number of clear aligner trays. The ellipsoid attachment is the best type of attachment because the resulting force is substantial and evenly distributed.

## Introduction

The rising interest in cosmetic dentistry in today's culture makes orthodontics necessary. One of the most common operations in cosmetic dentistry is orthodontic treatment, which is done to straightens the teeth to provide a healthy occlusion and an aesthetically pleasing appearance. 1 2 Clear aligner treatment (CAT), which was introduced to the market in the late 1990s, has become an important aspect of orthodontics. CAT has gained popularity among orthodontists and patients due to its comfort. In contrast to typical fixed orthodontic appliances, clear aligners are removable and clear; hence, patients may select CAT for aesthetic reasons. 3 Improved patient acceptability and a high quality of life are the primary benefits of CAT. Compared with conventional fixed braces, CAT is less painful and more effective in improving periodontal tissue health and minimizing apical resorptions, dental trauma, and microbial risk. 4 Although CAT may be desirable to patients, its efficacy is difficult to determine compared with conventional orthodontic appliances. According to a systematic review, CAT may be successful for some types of orthodontic malocclusions, such as aligning and leveling arches and controlling anterior intrusion, posterior buccolingual inclination, and upper molar bodily movements, but not for others, including rotational tooth correction and anterior buccolingual inclination. 5 Rotations, extrusions, and torque movements are extremely difficult to execute with clear aligners, though they can be predicted with a high degree of certainty for the leveling and aligning, intrusion, and bodily distalization of upper molars of less than 1.5 millimeters. 6 7 Furthermore, patient compliance is a crucial factor in the success of CA treatment. 8 Interproximal reduction, interarch elastics, attachments, and other orthodontic auxiliaries are commonly used in conjunction with CAT, thus expanding the range of malocclusions that can be corrected with aligners. 6 9

Composite attachments—tiny composite buttons with a predetermined geometry that are bonded to the surface of the teeth—may be used to improve the retention of clear aligners. Attachments offer retention, help the transmission of force from clear aligners to the teeth, and allow for sophisticated tooth movements, such as translation. 10 Yokoi et al 11 used finite element analysis (FEA) to study the impact of attachments on maxillary dental diastema closure and found that attachments allow for bodily movement. Another FEA investigation revealed that variations in attachment form and position have no effect on forces and the amount of tipping movements during space closure. 12 Kim et al 13 compared the best shape and position of canine attachments for extrusion, intrusion, torque, and rotation and found that a cylinder-shaped, lingually placed attachment is the best for tooth movement. Costa et al 14 tested three extrusion attachments, namely, modified ellipsoid, beveled, and rectangular attachments with prominence and an inclined plane at the vestibular side to increase active surface. They found that the shape of attachment can affect force intensity and direction, and the modified ellipsoid attachment without an edge and less prominence shows superior mechanical function despite producing less extrusive force.

In addition to attachments, the accuracy of orthodontic treatment with CAT is influenced by other factors, such as aligner thickness. To minimize the duration of orthodontic treatment, Align Technology recommends determining the proper thickness of the aligner according to the desired amount of tooth movement and the form and position of attachments according to the desired amount and direction of tooth movement. The thickness of the aligner is associated with the amount and duration of the orthodontic force applied to the tooth and is a significant variable in developing an orthodontic treatment plan while considering the periodontal ligament (PDL). 15 16 Patients with bimaxillary protrusion benefit from orthodontic therapy, which typically involves the elimination of premolars and the retraction of anterior teeth. 17 Although attachments have a high potential for tooth movement and aligner thickness affects aligner accuracy, the biomechanical properties of attachments are poorly understood. Hence, making a proper choice in dental practice cases is difficult. To the best of our knowledge, no study has assessed the biomechanics of various attachment designs and aligner thicknesses for anterior retraction that allow for effective movement with clear aligners. Instantaneously following force loading, FEA may determine the initial movement of teeth. This method has been extensively utilized in an orthodontic biomechanics research to analyze the stress response of external forces in complex structures and is a useful tool for simulating tooth displacement patterns in orthodontics. 18 Therefore, this work uses FEA to examine the stress distribution generated by various composite attachment designs and aligners thicknesses during anterior retraction with clear aligners.

This study hypothesizes that the thicker the clear aligner, the greater the force applied to orthodontic tooth movement. If the clear aligner continues to get thicker, then it will exert an excessive amount of orthodontic force, which will result in a range of undesirable side effects. Another hypothesis is that different attachment configurations forms would provide different orthodontic forces. The ellipsoid attachment is the best attachment for anterior retraction because the resulting force is normally distributed in all areas.

## Material and Methods

### Finite Element Model Preparation

This research was approved by the ethics committee of the Faculty of Dentistry, UGM with the number No.085/KE/FKG-UGM/EC/2022. An adult male participant with Class II Division 1 angle malocclusion had his teeth and maxillary bone remodeled using cone-beam computed tomography (CBCT) scanning. Another subject had a proclined upper incisor to NA line (U1-NA) of 40.99 degrees with premolar extraction indication and no bone structure abnormalities. A CBCT imaging machine (Genoray, Korea, 60 kVp, 60 mAs) was used to obtain a dental model of the patient, which was then reconstructed in FEA. The tooth elements, PDL, palatal bone, and maxillary alveolar bone were assembled together to produce a 3D model. Under the assumption that human upper and lower dentitions are symmetrical, only half of the dentition (half-arch) was employed in this investigation.

This study focused on central incisor (U1) elements with proclination that require an anterior retraction force to correct the inclination of the tooth. With the cortical bone's thickness set at 2 mm, the mandibular alveolar bone was divided into cortical and cancellous bones. PDLs were generated as 0.2 mm offsets between the teeth and alveolar bone using the three-dimensional (3D) modeling software SolidWorks. Four node tetrahedral elements were applied to mesh the resulting 3D structures that comprised the dental FE model in ANSYS 2020R2 software, a commercially accessible FEA software program.

For analysis, aligners were divided into five groups according to their thickness: 0.75, 0.85, 0.95, 1.05, and 1.15 mm. For the study of attachment types to be attached to the U1 and the effect of the force for anterior retraction, various attachments were divided into three groups: rectangular attachments, ellipsoid attachments, and pyramidal attachments. All the attachments were inserted in the central area of the tooth central incisor (U1) using the mean point between the incisal edge and cementoenamel junction. The three attachment configurations have almost the same dimension (width × height of 3 mm × 4 mm) and prominence (1.5 mm). After the installation of aligners with various thicknesses and attachments with various shapes was simulated, the force was applied backward and perpendicular to the axis of symmetry of the teeth (parallel to and opposite to the Z+ axis).

FEA meshing or discretization was accomplished by converting a continuous solid domain into a discrete computational domain with a finite number of elements so that structural equations can be computed numerically using FEA. Owing to its advantage of generating complicated geometries, tetrahedral mesh was used to connect complex areas, such as indentations and details. The fixed support was established by limiting the translational and rotational movement of the alveolar bone in the x, y, and z directions. In this case, the fixed support position was at the top of the alveolar bone. In the simulation, a fixed type boundary condition was used around the bone (in blue) to keep the degree of freedom (DOE) of the bone unchanged. The mesh was adjusted to obtain accurate FEA simulation results. Initially, the global mesh was set to 2 mm. The teeth and PDL layers were adjusted by 1 and 0.3 mm, respectively. Afterward, the alveolar bone was adjusted by 1.5 mm and then used as a rigid body in the computation of the stress on the PDLs, which were designed as hyperelastic materials to approximate their genuine mechanical properties. Finally, the clear aligner and attachment were adjusted by 1 and 0.2 mm, respectively. The properties of each of these materials are listed in Table 1 .

**Table 1:** Material properties used in the finite element model

Component	Young's modulus (MPa)	Poisson's ratio
Teeth	1.96 × 10 ^4^	0.30
Periodontal ligament	3 × 10 ^2^	0.30
Alveolar bone	1.37 × 10 ^3^	0.30
Clear aligner	528	0.36
Attachment	12.5 × 10 ^3^	0.36

## Results

**Fig. 1:**
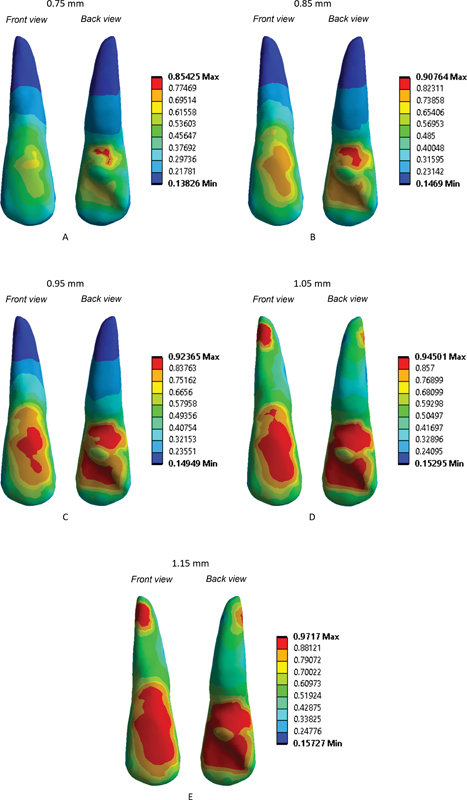
. Finite element analysis simulation results for aligner thicknesses of 0.75, 0.85, 0.95, 1.05, and 1.15 mm.

The FEA simulation results were in the form of pressure contours on the simulated tooth model. Simulation analysis was conducted to compare five variations of aligner thickness and three attachment configurations. Data were analyzed visually and numerically from von Mises stress in each region such as the crown, root, and PDL. FEA results for aligner thickness show that an increase in the clear aligner thickness affects the amount of stress experienced by the teeth. Fig. 1 shows the finite analysis results from the pressure contours. Visual analysis reveals areas of the teeth and PDL with varying map colors. The blue shows the light stress in that area, the green shows the medium stress, and the red shows the enormous stress.

According to the above FEA results on the contour, the greater thickness of the aligner, the higher the stress experienced by the teeth. The aligner with 0.75 mm thickness shows a minimum stress value of 0.13826 MPa and a maximum stress value of 0.85425 MPa. Visual observation reveals blue areas at the root, green and yellow areas at the crown, and slight red area at the cervical palate. When the aligner with thickness of 0.85 mm was used, the minimum and maximum stress increased to 0.14690 and 0.90764 MPa, respectively. Visual observation revealed many yellow and orange areas at the crown. When the aligner with thickness of 0.95 mm was used, the minimum and maximum stress increased to 0.14949 and 0.92365 MPa, respectively, with many orange and red areas appearing at the crown. When the aligner with thickness of 1.05 mm was used, the minimum and maximum stress increased to 0.15295 and 0.94501 MPa, respectively. Visual observation revealed many red areas at the crown and root tooth. When the aligner with thickness of 1.15 mm was used, the minimum and maximum stress increased to 0.15727 and 0.97170 MPa, respectively. Visual observation revealed a wide red area at the crown and root tooth. The results indicated that the best aligner thickness is between 0.75 and 0.95 mm. Meanwhile, the aligners with thickness more than 0.95 mm give excessive force, which is characterized by many red areas and will impact the condition of the teeth, such as tooth root resorption and periodontal tissue destruction.

**Fig. 2:**
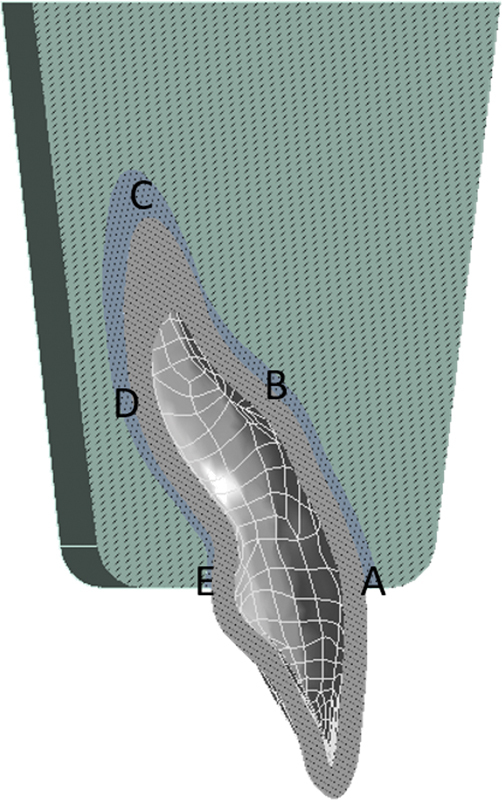
Division of the four areas of the tooth structure affected by the force of the clear aligners: A–B (cementoenamel junction labial–1/3 root labial), B–C (1/3 root labial–root apex), C–D (root apex–1/3 root palatal), and D–E (1/3 root palatal–cementoenamel junction palatal).

**Fig. 3:**
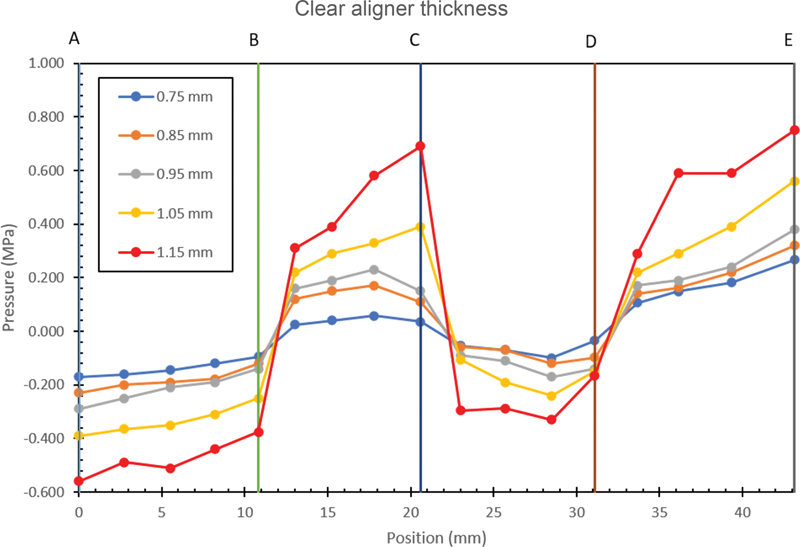
Stress distribution in the tooth structure's four areas based on the force of each clear aligner thickness.

The amount of stress experienced by the crown of the tooth attached to the aligner is transmitted in the cervical direction of the tooth due to the force exerted by the aligner. Therefore, investigating several points in the area is necessary. Stress was investigated in four areas to determine which area of the tooth was subjected to the greatest stress. The investigation area was divided into four areas: A–B, B–C, C–D, and D–E, and each part was analyzed to determine the greatest force exerted by the aligner ( Fig. 2 ). The magnitude of the stress experienced by the four areas can be seen from the graph in Fig. 3 .

Fig. 3 illustrates the amplitude of the initial stress acting at different points of the PDL as a function of clear aligner thickness. For all the aligner thicknesses, tensile stress was generated between regions A–B and C–D, and compressive stress was formed between regions B–C and D–E. The graph shows that the clear aligners with thicknesses of 0.75, 0.85, and 0.95 mm induced stable and constant tensile and compressive stresses around 0.200 MPa to 0.300 MPa. With the increase in the thickness of the appliance, the intensity of the tensile and compressive stresses also increased. Meanwhile, a difference was observed between the aligners with thicknesses of 1.05 and 1.15 mm. The clear aligner with 1.05 mm thickness induced significantly increased tensile and compressive stresses reaching 0.400 MPa to 0.500 MPa, and the clear aligner with 1.15 mm thickness induced extremely large tensile and compressive stresses reaching 0.600 MPa to 0.700 MPa.

**Fig. 4:**
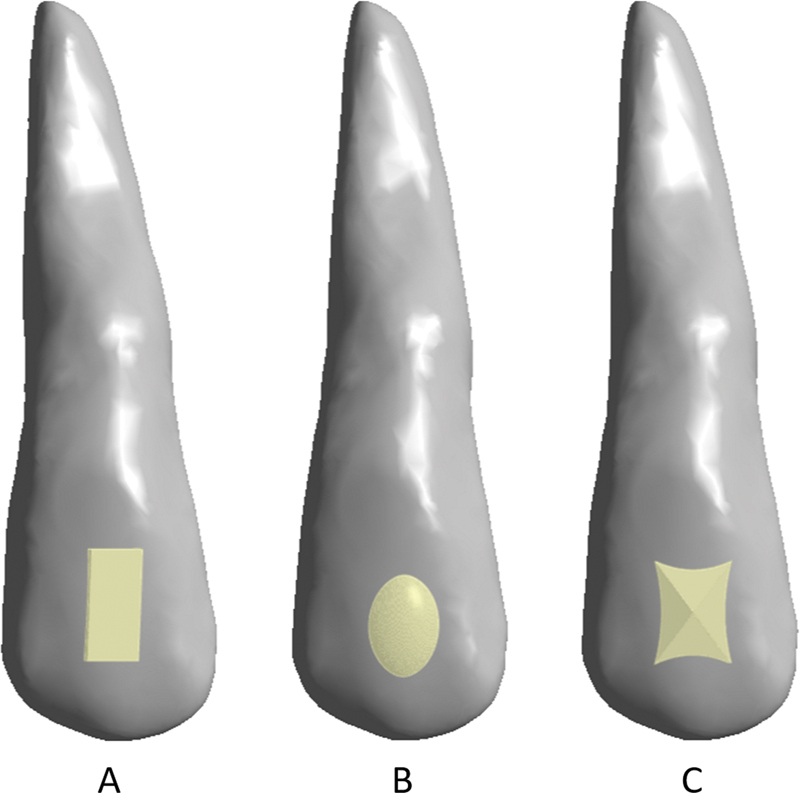
Configuration of incisor geometry (U1) with attachments: ( **A** ) rectangular attachment, ( **B** ) ellipsoid attachment, and ( **C** ) pyramidal attachment.

After the FEA for the thickness of the aligners, a comparison was conducted on three attachment configurations, namely, rectangular attachment, ellipsoid attachment, and pyramidal attachment. Setup attachment configuration FEA can be seen from the graph in Fig. 4 .

**Fig. 5:**
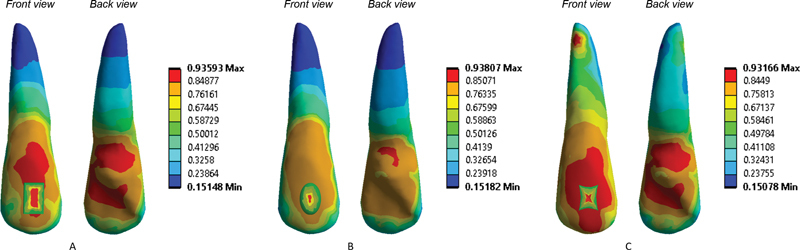
Finite element analysis simulation results rectangular attachment (A), ellipsoid attachment (B), and pyramidal attachment (C).

FEA of the three attachment configurations showed the different Von mises maximum and minimum stress ( Fig. 5 ). The rectangular attachment had a minimum stress of 0.15148 MPa and a maximum stress of 0.93593 MPa; visual observations revealed blue areas at the root and many red areas at the crown. The ellipsoid attachment had a minimum stress of 0.15182 MPa and a maximum stress of 0.93807 MPa; visual observations revealed blue areas at the root and orange areas at the crown. The pyramidal attachment had a minimum stress of 0.15078 MPa and a maximum stress of 0.93166 MPa; visual observations revealed a green area and small red area at the root and many red areas at the crown. These results showed that even when clear aligners with different attachment configurations are subjected to the same force pressure, the distribution of these forces on various tooth surfaces and tooth roots will vary. The distribution of forces on ellipsoid attachments is evenly distributed.

## Discussion

Despite the expanding global demands for clear aligners as malocclusion orthodontic treatment, questions remain about their effectiveness in achieving complex tooth-controlling movements. This phenomenon could be related to a lack of clarity on aligners' force/moment-transmission system. In current orthodontic treatment, the use of clear aligners in orthodontic treatment still needs improvement because they are unable to provide a large force. This study proposes an alternative solution to solve this problem, that is, to increase the usual thickness of the aligner (0.75 mm) and use attachments so that the force effectively acts on a tooth for orthodontic treatment. Variations in aligner thickness and attachment types were analyzed using FEA and were visually and numerically obtained from von Mises stress. 11 In the orthodontic field, FEA is commonly used to evaluate orthodontic appliances, determine the stress–strain distribution in the periodontium, and simulate orthodontic tooth movement. In addition to clear aligner biomechanics, FEA results provide adequate accuracy in predicting clinical outcomes. 19 20 Visual analysis reveal the areas of the teeth and PDL with varying map colors. The blue color shows the light stress in that area, the green shows the medium stress, and the red shows the enormous stress. 13

Analysis of aligner thickness revealed that increasing the thickness of the clear aligner will also increase the minimum and maximum von Mises stress. Visual analysis showed that increasing the thickness of the aligners will also increase the area of force applied to the tooth structure and roots as characterized by the color changes. When the aligner with 0.75 mm thickness was used, blue areas were found at the root, and green and yellow areas at the crown, and a slight red area at the cervical palate. According to Seo et al, 21 transparent aligners with thicknesses of 0.5 and 0.75 mm can transfer enough pressure to the PDL for orthodontic treatment and correction, and the stress deformation values evaluated are well within the range to cause a dramatic change in the PDL. 21 22 When a 0.85 mm thick aligner was utilized, multiple yellow and orange spots developed at the crown. This conclusion suggested that the force applied to the tooth's crown is sufficient and equally distributed throughout the entire area. Once the 0.95 mm thick aligner was employed, a red area was discovered on the tooth's crown, suggesting that the force exerted to the crown was significant (too large). Therefore, aligners of 0.95 mm thickness must be used with caution because their prolonged application can damage the structure of the tooth crown. Many red areas occurred at the crown and root tooth when the aligner with a thickness of 1.05 mm was employed, indicating that the force exerted to the crown is significant and may cause damage to the dental crown structure and root resorption. When the aligner with 1.15 mm thickness was used, a wide red area was found at the crown and root of the tooth, indicating that the force applied to the crown and periodontal tissue is too large. This problem can lead to a variety of unpleasant side effects, including damage to the tooth crown structure, root resorption, and deterioration of the periodontal tissue. When excessive force is applied, there is a greater chance that indirect bone resorption will occur rather than direct bone resorption; as a result, the speed at which the teeth move will be decreased. 23 The results indicated that the best aligner thickness is between 0.75 and 0.85 mm. Aligners with 0.95 mm thickness can still be used in certain cases that require a large tooth movement force but not for a long period of treatment and only in a few clear aligner trays. The prolonged use of an aligner this thick could harm the dental crown structure. Furthermore, when aligners with thickness of 1.05 and 1.15 mm or above are used, the tooth crown structure may be harmed, leading to root resorption, destruction of periodontal tissues, and high possibility of attachment detachment. 24

This research investigated several points in the area of the tooth to determine which was subjected to the greatest stress. Under all the aligner thicknesses, compressive stress was observed between areas B–C and D–E and tensile stress was found between regions A–B and C–D. After applying the translational force by the clear aligner, the impact of the force on several points areas of the tooth is analyzed on the graph. The graph demonstrates that the tensile and compressive stresses for the clear aligners with 0.75, 0.85, and 0.95 mm thickness were stable and constant. However, the aligner with 1.05 mm thickness showed different results, that is, the tensile and compressive stresses improved dramatically. The clear aligner with 1.15 mm thickness demonstrated extremely high tensile and compressive values. The graph supported previous studies, which stated that for aligners with thickness of 1.05 and 1.15 mm or above, the tensile and compressive stresses drastically increase, are too large, and may cause damage to the crown structure and periodontal tissues. 24

After the FEA for aligner thickness, the three attachment configurations were compared. Attachments may be used to improve the retention of clear aligners. Attachments offer retention, help the transmission of force from clear aligners to the teeth, and can support clear aligners to correct various teeth movements such as correction of crown tipping, axial rotation, and tooth translation. 25 The three types of attachments subjected to FEA simulation were rectangular attachment, ellipsoid attachment, and pyramidal attachment. The results showed that the minimum and maximum von Mises stress did not significantly differ among the three attachment configurations. The finding of visual analysis can be described as follows. When the rectangular attachment was used, blue areas at the root and many red areas at the crown were found. When the ellipsoid attachment was used, blue areas at the root and orange areas at the crown were observed. When the pyramidal attachment was used, a green area and a small red area at the root and many red areas at the crown appeared. Therefore, when clear aligners with different attachment configurations are subjected to the same force pressure, the forces' distribution on different dental surfaces and tooth roots will differ. 26 This study demonstrated a more uniform distribution of forces on ellipsoid attachments. The findings are in agreement with Costa et al, 14 who explained that the type of attachment in the form of an ellipsoid/cylinder has the best mechanical performance than other types of attachments. In addition, Kim et al 13 stated that the desired stress distribution can be achieved properly using a cylinder attachment.

Given that we only studied the movement of a single incisor tooth, our study cannot accurately reflect the actual orthodontic treatment scenario. In addition, situations that may result from masticatory movements and masseter muscles after using aligner devices were not evaluated. Therefore, future preclinical evaluation and biomechanical research are necessary to determine the efficacy of multiple tooth movement in actual orthodontic treatment conditions. Additional study is also required to improve the effectiveness of orthodontic treatment by analyzing the relationship between masseter muscle fiber properties and malocclusion following the use of clear aligners.

## Conclusion

This research shows that 0.75 to 0.85 mm is the ideal aligner thickness for anterior retraction. An aligner with 0.95 mm thickness can still be used when a significant amount of tooth movement force is needed; however, this exception is only applicable in a limited number of clear aligner trays. Configuration analysis reveals that an ellipsoid attachment is the best type of attachment for movement in anterior retraction because the resulting force is substantial and normally distributed in all areas.
